# Successful Delayed Interval Delivery in Twin Pregnancy After Early Premature Rupture of Membranes of a Leading Fetus: A Case Report

**DOI:** 10.3390/medicina60111800

**Published:** 2024-11-02

**Authors:** Won-Kyu Jang

**Affiliations:** Department of Obstetrics and Gynecology, Daegu Dongsan Hospital, Keimyung University School of Medicine, 56, Dalseong-ro, Jung-gu, Daegu 41931, Republic of Korea; cindeln@naver.com

**Keywords:** delayed delivery, twin pregnancy, cervical cerclage, premature rupture of membranes

## Abstract

A 36-year-old primigravida conceived dichorionic diamniotic twins via in vitro fertilization. The first twin experienced premature rupture of membranes at 18 weeks and 5 days of gestation. Despite antibiotic treatment, the first fetus suffered intrauterine fetal death and was delivered three days later at 19 weeks and 1 day. Using ritodrine, the remaining umbilical cord was repositioned, and an emergency cerclage was performed. Ritodrine was continuously administered post-surgery but discontinued at 24 weeks and 3 days due to improved uterine contractions. The patient later delivered a healthy baby via cesarean section at 38 weeks due to decreased fetal movement and breech presentation. This rare case of premature rupture of membranes in one of the twins before the viability term, followed by a successful delayed delivery, is presented here.

## 1. Introduction

Twin pregnancies have a higher incidence and risk of complications, of which preterm premature rupture of membranes (PPROM), defined as the spontaneous rupture of membranes before 37 weeks of pregnancy, is the most notable and accounts for approximately one-third of all preterm births [1]. Most cases of premature rupture of membranes (PROM) in twin pregnancies are a complication of very early rupture of the membranes, resulting in the spontaneous delivery of both fetuses after a short latent period [2]. In a twin pregnancy, the second twin being delivered late after the first fetus is very rare.

Delayed interval delivery occurs when one fetus in a multiple pregnancy is delivered, while the other fetus remains in utero and is not born immediately afterward [3]. Case reports have shown that delayed interval delivery can be successfully achieved in selected cases, although the optimal management approach remains unclear [4,5,6]. Although various treatment methods have been suggested, such as long-term bed rest, broad-spectrum antibiotics, corticosteroids, tocolytics, and cervical cerclage, there is no standardized protocol for managing delayed interval deliveries [7,8,9].

We here present a case of PPROM in one of the dichorionic diamniotic twins before the viable term. Following delivery of the affected fetus, we performed simultaneous cervical cerclage, and following appropriate tocolytic and antibiotic administration, we successfully extended the pregnancy of the remaining fetus and delivered a healthy baby.

## 2. Case Report

**Figure 1:**
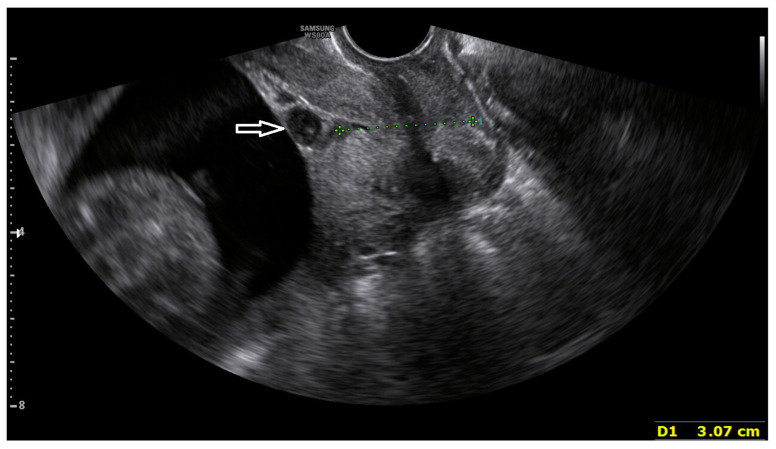
Transvaginal ultrasonography showing a cervix after emergency cervical cerclage. The arrow indicates the remaining umbilical cord of the first fetus delivered, which was pushed into the cervix during the cerclage procedure.

A healthy 36-year-old primiparous woman with no comorbidities became pregnant with dichorionic diamniotic twins through in vitro fertilization and embryo transfer and underwent regular prenatal checkups at the outpatient clinic of the Obstetrics and Gynecology Department of our hospital at 13 weeks and 1 day of gestation. At 18 weeks and 5 days of gestation, she visited the emergency department of our hospital, owing to leakage sensation and lower abdominal pain and underwent examination. An ultrasound examination revealed the absence of amniotic fluid around the first fetus. The fetal heart rate was 121 beats/min, and uterine contractions were detected at 1–2 min intervals using a nonstress test (NST). Fever was not noted during the visit; however, initial laboratory examinations revealed the following results: white blood cell, 13,710/uL; neutrophils, 78.6%; and C-reactive protein (CRP), 0.7 mg/dL. Subsequently, the following prophylactic broad-spectrum antibiotics were started: (1) intravenous (IV) ceftriaxone at a dose of 2 g every 24 h, (2) IV metronidazole at a dose of 500 mg every 8 h, and (3) oral clarithromycin at a dose of 500 mg every 12 h. On observation, the first fetus developed intrauterine fetal death, and 3 days later, at 19 weeks and 1 day of gestation, this fetus was delivered. The fetus weighed 230 g. The mother remained afebrile; however, her CRP level increased to 2.2 mg/dL, with white blood cell and neutrophil counts of 11,800/uL and 86.1%, respectively, and she continued to experience lower abdominal pain. Using Vicryl 1-0, the umbilical cord, which was exposed outside the cervix, was tied with a suture approximately 5 mm from the cervix and cut with scissors 10 mm away from the cervix. Subsequently, 100 mg of ritodrine was mixed with 500 mL of 5% dextrose solution and administered intravenously at a rate of 16 cc/h. While maintaining the ritodrine dose, the remaining umbilical cord was repositioned into the cervix using Kelly Placenta Forceps, and an emergency cervical cerclage was performed using the McDonald cerclage technique under spinal anesthesia (Figure 1). The antibiotics mentioned earlier were started 3 days prior to the surgery and continued for an additional 14 days postoperatively.

**Figure 2:**
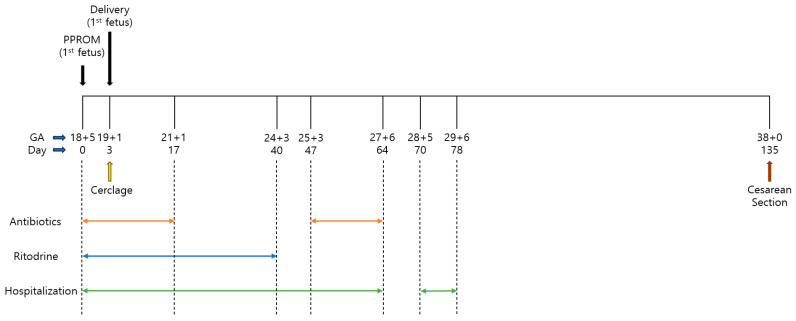
Timeline of delayed interval delivery in a twin pregnancy following early premature rupture of membranes in the leading fetus. GA—gestational age; PPROM—preterm premature rupture of membranes.

Ritodrine was continuously administered postoperatively, with the infusion rate increased by 16 cc/h at 15 min intervals, up to a maximum of 80 cc/h. If the maternal heart rate exceeded 120 beats per minute, the dose was reduced by 8 cc/h and maintained for 12 h until uterine contractions ceased completely. However, contractions regularly recurred every time ritodrine was stopped; therefore, the same protocol was performed five times. Furthermore, at 20 weeks and 5 days of gestation, the medical staff and the patient agreed that IV ritodrine could not be discontinued; therefore, continuous ritodrine administration was decided and maintained at the minimum dose that could control contraction. At 24 weeks and 3 days of gestation, after 26 days of continuous use, ritodrine was discontinued due to no changes in cervical length, despite the presence of irregular contractions occurring 3–4 times per hour. However, at 25 weeks and 3 days of gestation, extended-spectrum beta-lactamase-positive Escherichia coli was identified in the cervical culture; therefore, IV meropenem 1 g three times daily was started. At 27 weeks and 6 days of gestation, the patient was discharged without any untoward symptoms, having remained in the hospital for a total of 64 days, 61 days after the delivery of the first fetus. Subsequently, she was hospitalized once at 28 weeks and 5 days of gestation owing to vaginal spotting and was discharged at 29 weeks and 6 days of gestation, without tocolytic administration. Since then, she has been followed up as an outpatient without further hospitalization and has remained asymptomatic. The fetus was in breech presentation, so a cesarean section was scheduled for 38 weeks and 3 days of gestation. However, at 38 weeks, the mother reported decreased fetal movement and anxiety, prompting an earlier cesarean delivery. This occurred 135 days after the rupture of the first fetus’s membranes. The delivered baby, a healthy female weighing 2970 g, had an Apgar score of 9/10 (Figure 2).

**Figure 3:**
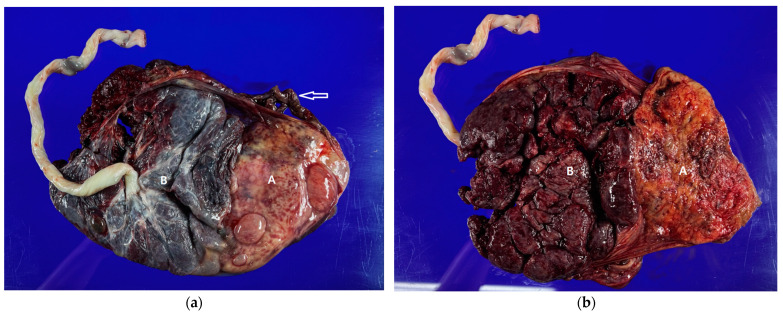
Postpartum gross findings of the two placentas: (**a**) Fetal surface of the placentas, showing (A) the placenta of the first fetus, (B) the placenta of the second fetus, and the arrow indicating the umbilical cord of the first fetus; (**b**) Maternal surface of the two placentas.

Culture results did not show any pathogenic bacteria in the stitched-off cervical umbilical tape. Placental biopsy results revealed no significant findings, including chorioamnionitis, funisitis, or villitis, other than an extensive infarction in the placenta of the first fetus; no significant findings were observed in the placenta of the second fetus (Figure 3).

## 3. Discussion

Owing to the advancement and widespread use of artificial reproductive technology, the incidence of twin pregnancies is increasing, and perinatal complications occur more frequently than with singleton pregnancies. In the United States, the incidence of PROM in twin pregnancies is 7.1% [10]. Of these cases, 6.1% were associated with the delivery of a second fetus being delayed by at least 1 week after the delivery of the first fetus, and few of these cases survived to the full term of pregnancy. The most common reasons for preterm delivery are preterm labor and chorioamnionitis. To our knowledge, there have only been two successful cases of full-term delivery after 37 weeks of gestation. Hamersley et al. reported the longest delayed delivery duration of 153 days, with the first baby born at 15 weeks and 3 days of gestational age and the second baby born at 37 weeks and 2 days of gestational age [11]. Petousis et al. reported a delayed delivery duration of 141 days, with the first and second babies born at 17 weeks and 2 days of gestational age and 37 weeks and 2 days of gestational age, respectively [12]. Our case is one of the few successful cases worldwide wherein the fetus was successfully delivered to the full term of pregnancy through delayed interval delivery, and the delayed delivery duration of 135 days is considered highly significant.

One of the most controversial issues among recommended treatments for delayed interval delivery in multiple pregnancies is prophylactic cervical cerclage [13]. However, one study of delayed interval delivery concluded that performing cervical cerclage immediately after delivery of the first fetus significantly prolonged the interval between the deliveries of twins without increasing the risk of intrauterine infection [5]. Additionally, cervical cerclage minimizes exposure of the fetus’ amniotic membranes to bacteria in the vagina and stabilizes the cervix [14]. In our case, no findings of infection in the cervical culture results following cervical cerclage were observed, and no findings of chorioamnionitis in the placental biopsy performed following delivery were noted, indicating good results of delayed interval delivery. Moreover, the use of appropriate broad-spectrum prophylactic antibiotics played a significant role. Oh et al. reported that the administration of antibiotics (ceftriaxone, clarithromycin, and metronidazole) for treating intra-amniotic infection and inflammation resulted in the resolution of intra-amniotic inflammatory process or intra-amniotic infection in 75% of patients and that approximately 60% of these cases were associated with treatment success [15]. Similarly, we administered broad-spectrum antibiotics, including ceftriaxone, clarithromycin, and metronidazole, for two weeks following the cerclage procedure. As a result, we achieved favorable outcomes, with no specific findings of chorioamnionitis or intra-amniotic infection.

Considering PPROM alone, current data are insufficient to support the use of tocolytics for suppressing uterine contractions in women with PPROM, as most clinical guidelines acknowledge [16,17]. However, for delayed interval delivery in multiple pregnancies, previous cases have suggested that prescribing tocolytics when uterine contractions occur even after the birth of the first fetus is necessary [18]. As in previous studies, we also used tocolytics very actively by carefully monitoring uterine contractions. Although no specific inflammatory findings were observed, uterine contractions recurred whenever tocolytics were stopped. Therefore, tocolytics were actively administered despite there being no changes in the cervical length; eventually, the recurrent uterine contractions were controlled. We believe that actively using tocolytics is also a factor in successful outcomes.

## 4. Conclusions

The treatment policy for delayed interval delivery of PROM in twin pregnancies is not well established. However, as in some cases and our case wherein a successful delayed interval delivery was achieved, pregnancies are maintained by active treatment. Considering the maternal condition and the patient’s intention to continue the pregnancy, sufficient counseling on pregnancy complications, including premature birth, should be provided. We hope that our case will help in clinical decision making and patient counseling for patients in similar situations.

## Data Availability

Data are contained within the article.
